# Technical Quality and Students' Perception of Endodontic Preclinical Training Using Natural or LikeReal Artificial Teeth

**DOI:** 10.1111/eje.70024

**Published:** 2025-07-28

**Authors:** Gabriela Biagioni, Fernanda Comodo, Marcelo Santos Coelho

**Affiliations:** ^1^ São Leopoldo Mandic School of Dentistry Campinas São Paulo Brazil

**Keywords:** dental education, endodontics, replicas, undergraduate

## Abstract

**Introduction:**

This study evaluated and compared the technical quality of treatments performed during the preclinical training using artificial and natural teeth and the students' perceptions regarding their learning process with the 2 groups.

**Materials and Methods:**

The study assessed the teeth used for preclinical training by 2nd‐year students at São Leopoldo Mandic School of Dentistry. The natural teeth group (NTG) included the teeth used by the class of 2021, and the artificial teeth group (ATG) assessed the artificial teeth (LikeReal, Porto Alegre, Brazil) used by the class of 2022. The teeth were visually and radiographically observed, and the errors were divided according to the location in the crown or in the root. Errors in the crown included perforation, damage to the marginal ridge, fracture, and remnants of gutta‐percha. Errors in the roots included overfilling, obturation at the apex, obturation > 2 mm short of the apex, voids, instrument separation, ledge, and root fracture. The students' perceptions were assessed by a questionnaire with 10 questions involving ethical, biohazard, technical challenges, and fairness of the evaluation. The chi‐squared test was used for differences at *p* < 0.05.

**Results:**

408 teeth were assessed, 204 in each group. In the NTG, remaining gutta‐percha, 36 (17.64%); voids, 53 (25.98%); and root filling at the apex, 48 (23.52%), were the most common errors. In the ATG, remaining gutta‐percha, 45 (22.05%); voids; and filling > 2 mm short, 65 (31.86%) were the most common errors. In the ATG, the occurrence of damage to the marginal ridge and filling > 2 mm short of the apex was less frequent than in the NTG; ledges and root fractures were more frequent in the ATG (*p* < 0.05). Regarding students' perception, NTG was superior in access and instrumentation; both groups thought that training with natural teeth was better. The students in the ATG were more concerned with the ethical and biohazard aspects.

**Conclusions:**

The technical quality of the treatments was similar in both groups; however, artificial teeth were more prone to fracture, indicating that improvements are necessary for the material. The students' perception was that natural teeth are more appropriate for their training.

## 1 Introduction

Preclinical endodontic training is essential for students to acquire the requisite skills before undertaking clinical procedures. Natural teeth have been served to this purpose for many years, presenting both advantages and drawbacks [1]. Artificial methods using resin blocks were initially proposed as an alternative to natural teeth in both undergraduate and graduate training [2]. Afterwards, these blocks were improved and were extensively used for research in various manners [3, 4].

With the emergence of micro‐CT technology, artificial teeth can now be fabricated by scanning and printing natural teeth using various materials [5]. These artificial teeth can better recreate anatomic and textural aspects of natural teeth and have been used for endodontic training [6]. These teeth can replace natural teeth, filling the gaps of ethical aspects of obtaining natural teeth. From an educational perspective, the use of these fabricated teeth facilitates standardisation and enables more precise assessment of students' performance.

The brand LikeReal Teeth (Like Real, Porto Alegre, Brazil) is a 3D printed tooth based on micro‐CT scans of natural teeth. It combines a patent pending technique mixing resin with ceramic fragments. According to the manufacturer, these teeth achieve a hardness similar to that of dentin. Moreover, the radiopacity of the teeth is similar to that of natural teeth. To the best of our knowledge, there is no study in endodontic literature evaluating these teeth for preclinical training.

Students' perceptions have been assessed concerning instrumentation techniques and overall challenges encountered during endodontic training [7]. It is important that their training not only enhances their skills, but also makes them confident when performing treatments in a clinical setting [8]. Ideally, the preclinical training should be as close as possible to the clinical scenario [9].

This study aimed to compare the number of errors made by undergraduate students using either natural or artificial teeth during their preclinical training. Additionally, this study's aim was to compare students' perceptions regarding the similarity of the training using natural or artificial teeth when compared to the clinical treatments. The null hypotheses tested were as follows: (1) the number of errors is similar between the groups; (2) students' perceptions are similar in both groups.

## 2 Materials and Methods

This study encompassed the evaluations of the natural or artificial teeth treated by undergraduate dental students during their preclinical training. Also, a questionnaire with 10 questions was sent to the students of both classes. The different steps of the study were reviewed and approved by the ethical committee of our institution (51171321.8.0000.5374; 51167621.5.0000.5374 and 52340421.8.0000.5374).

After the completion of the endodontic preclinical training, the teeth used during the activities, the charts, and radiographs remain in possession of the Endodontic department. The natural teeth from the 45 students of the Class of 2021 (Group NTG, *n* = 204) and the artificial teeth of the 31 students of the Class of 2022 (Group ATG, *n* = 204) were assessed. In the NTG, the students were required to provide the natural teeth for the training. This requirement did not mention any specific tooth type; however, the training for each student was the same, meaning that at least one maxillary and one mandibular tooth were used (central incisor, lateral incisor, canine, first and second premolar, first and second molar). Thus, the students made exchanges among them when deemed necessary. In the ATG, the students used the same teeth, mounted in a silicone arch including all left teeth of both mandibular and maxillary arches. The incisors, premolars, and maxillary second molar of the maxillary arch, as well as the central incisor, canine, premolars, and mandibular first molar of the mandibular arch were opaque. Meanwhile, the maxillary canine and maxillary first molar, mandibular lateral incisor, and mandibular second molar were translucent, so students could see the pulp chamber and root canal system.

The students were taught a classical approach for the pulp chamber access, a crown down reciprocating technique for root canal instrumentation using the X1 Blue files (MK Life Medical Products, Porto Alegre Brazil) under 1% sodium hypochlorite and Endo PTC, followed by a final irrigation with EDTA [10]. Root canal filling was performed with either passive cold lateral condensation or Tagger's technique [11]. The same training was delivered to both groups, including 160 h of lectures, seminars, live demonstrations, and practical activities.

The teeth in each group were selected by the evaluators without knowledge of the names or grades of the students. Care was taken to select the same number of teeth in each tooth type for both groups. Errors made by the students were anatomically divided into errors in the crown or errors in the root. The following were considered errors in the crown: perforation, access damaging marginal ridge and/or enamel bridge, coronal fracture, and remaining gutta‐percha in the crown. Errors in the root were registered when they occurred: overfilling, filling shorter than 2 mm, voids in the filling, instrument separation, ledge and root fracture.

Two evaluators, DDS candidates, trained and calibrated for this purpose, assessed the teeth using radiographs and visual inspection. The assessment of the teeth was part of the Final Paper required for the students' graduation. The inspection was done under illumination with naked eyes, and a microscope (Zeiss Pico, Carl Zeiss AG, Jena, Germany) was used when deemed necessary. For the assessment of the working length (WL), the presence of separated instruments or voids in the filling, a digital radiograph was used. The ruler of the radiograph software was used for the measurement of the distance from the root canal filling to the radiographic apex. A third evaluator, a member of the Endodontic faculty, confirmed all the assessments and refereed any disagreement between the evaluators. All errors were registered in a spreadsheet (Excel, Microsoft Co., Redmond, WA). The percentage of the errors was calculated on a tooth basis.

In addition to the errors, a questionnaire was sent to the students asking about the perceptions of the training. The questionnaire was sent to the students of the NTG (*n* = 45) and ATG (*n* = 31) only after they completed their clinical endodontic training. For each group, 10 questions were asked: 9 closed questions and 1 question asking for suggestions. Five out of nine questions were equal for each group, and the other four were as similar as possible.

The chi‐square test was used for statistically significant differences between the groups; the significance level of 5% was used.

## 3 Results

Overall, 408 teeth were assessed, 204 teeth in the NTG and 204 teeth in the ATG (Table 1).

**TABLE 1:** Tooth type distribution in each group.

Tooth type	ATG	NTG
Maxillary incisors	38	38
Maxillary canines	15	15
Maxillary premolars	43	43
Maxillary molars	14	14
Mandibular incisors	42	42
Mandicular canines	8	8
Mandibular premolars	21	21
Mandibular molars	23	23
Total	204	204

In the ATG, remaining gutta‐percha, 36 (17.64%); voids, 53 (25.98%), and root filling at the apex, 48 (23.52%), were the most common errors. In the NTG remaining gutta‐percha, 45 (22.05%), voids, 65 (31.86%), and filling > 2 mm short, 65 (31.86%) were the most common errors. In the NTG, the occurrence of damage to the marginal ridge and filling > 2 mm short to the apex was more frequent than in the ATG; ledges and root fractures were more frequent in the ATG (*p* < 0.05). The overall number of errors in each group is presented in Table 2.

**TABLE 2:** Total and percentage of errors in each group.

	Artificial teeth group		Natural teeth group	
	Total of errors	Percentage	Total of errors	Percentage
Coronal perforation	0	0.00%	0	0.00%
Access with damage	4	1.96%	24	11.76%*
Coronal fracture	0	0.00%	3	1.47%
Remaining gutta‐percha	36	17.64%	45	22.06%
Overfilling	2	0.98%	5	2.45%
Underfilled	23	11.27%	65	31.86%*
Filling at the apex	48	23.52%	38	18.63%
Voids	53	25.98%	65	31.86%
No‐filled canals	1	0.49%	8	3.92%*
Separated instruments	2	0.98%	8	3.92%
Ledges	17	8.33%	6	2.94%*
Root fracture	12	5.88%	2	0.98%*

*Note:* Asterisks (*) indicate statistically significant differences at *p* < 0.05.

Twelve students in each group responded to the questionnaire, meaning 26.67% of NTG and 38.7% of ATG. For the NTG, 50% of the students responded that the teeth were easy to obtain; in the ATG, 83.3% of the students responded that the teeth were easy to achieve (*p* > 0.05). Most of the students in the NTG found no risk in using these teeth; meanwhile, the majority of the students in the ATG responded that artificial teeth are safer to use. The NTG group responded more frequently than the ATG that their teeth properly simulated the clinical findings for access and instrumentation (*p* < 0.05); there was no difference in the students' perception for irrigation, root canal filling, and radiographs (*p* > 0.05). Overall, the students found that natural teeth are better for endodontic preclinical training (*p* < 0.05). Only three students in the NTG and one in the ATG gave suggestions about the training. Tables 3 and 4 show the response for each questionnaire.

**TABLE 3:** Responses obtained with the questionnaire sent to the students who used natural teeth for their training.

	Natural teeth group	
	Tooth bank	Donation from practices
1. Where did you obtain the teeth	7	4
	Yes	No
2. Was it easy to obtain the teeth?	6	6
3. During my training I was concerned with the risk of contamination from the teeth.	3	9
4. Was there any ethical issue in obtaining the teeth	1	11
5. Compared to my colleagues my evaluation was fair	10	2
6. I had technical difficulties related to the teeth during my training	11	1
7. Do you consider natural teeth ideal for preclinical training?	10	2
8. The ergonomics in the laboratory closely simulates the clinical setting	4	8
9. Did the training properly simulate the clinical setting in relation to the following aspects?		
Access	12	0
Irrigation	9	3
Instrumentation	11	1
Filling	10	2
Radiographs	5	7
10. Comments about your training	1. “I'd rather using natural teeth because they give us closer simulation to the difficulties we will face in clinic such as atresic canals…” 2. “Greatest challenge was to apply the techniques in a real patient with different ergonomics” 3. “Using human teeth give us na excelente perception of the anatomy and its variations (and that is what we face on a daily basis) it also gives a certain moral sense of respect with the specimens”	

**TABLE 4:** Responses obtained with the questionnaire sent to the students who used artificial teeth for their training.

	Artificial teeth group	
	Yes	No
1. Was it easy to obtain the teeth?	10	2
2. I could have obtained natural teeth if required to?	4	8
3. Artificial teeth are safer than natural teeth for preclinical training	11	1
4. Was there any ethical issue in obtaining the teeth	10	2
5. Compared to my colleagues my evaluation was fair	10	2
6. I had technical difficulties related to the teeth during my training	11	1
7. Do you consider artificial teeth ideal for preclinical training?	4	8
8. The ergonomics in the laboratory closely simulates the clinical setting	0	12
9. Did the training properly simulated the clinical setting in relation to the following aspects?		
Access	7	5
Irrigation	8	4
Instrumentation	4	8
Filling	8	4
Radiographs	7	5
10. Comments about your training	1. “more resistant teeth that do not heat or fracture”	

## 4 Discussion

This study aimed to compare natural and artificial teeth for endodontic preclinical training. Despite the use of two different classes, the training delivered for the students and technique applied were similar for both groups. Therefore, the errors found could be impacted by the teeth used and not by the training per se, and the results achieved are meaningful. The number of errors was different between the groups in some aspects; therefore, the null hypothesis 1 was rejected.

The results of the errors in the crowns found no perforation during the access of the teeth in both groups. This fact can be attributed to the good training the students received. Moreover, the hardness of the artificial teeth allowed the students to reach the pulp chamber without serious deviation from the path. Both groups had previous training with natural teeth during their restorative training delivered by other faculty. One can assume that a very soft material could result in perforation by the ATG students; therefore, the artificial teeth seem to properly replicate the natural ones in this aspect. Three coronal fractures occurred in the NTG and no fractures in the ATG. There was no control of the storage of the natural teeth, which can contribute to these fractures. The resistance of the artificial teeth seemed to be sufficient for a training without crown fractures. The higher percentage of errors in the crowns was associated with the presence of remaining gutta‐percha in the crown. As the percentage of this occurrence was similar in both groups (22.06% for NTG and 17.64% for ATG) we can conclude that both teeth were similar in this aspect. In this subject, more attention should be paid by faculty to emphasise the importance of the proper cleaning of the pulp chamber.

Only one aspect in the crown resulted in differences between the groups. The damage to the marginal ridge or to the enamel bridge was higher in the NTG than in the ATG. It is worthwhile to mention that only 1.96% of the teeth in the ATG presented this error, probably as a consequence of proper training on this aspect associated with good resistance of the teeth. However, the poor performance of the natural teeth should be carefully considered. As only final images of the teeth were considered, this result can be a consequence of severely damaged teeth prior to the access.

The undergraduate program of our institution recommends the filling 1 mm short from the radiographic apex as the ideal. According to Sjögren et al., filling from 0 to 2 mm from the apex presents better outcomes [12]. A recent clinical study showed 73.5% of adequate length in treatments performed by fourth‐ and fifth‐year dental students [13]. In the present study, there was little occurrence of fillings beyond the apex, 0.98% for ATG and 2.45% for NTG. Fillings at the apex were more frequent, but also with no difference between the two groups, 23.52% for ATG and 18.63% for NTG. As students are naturally concerned about grades, it is reasonable to infer that some overfilling was intentionally removed by the students, artificially increasing the number of fillings at the apex. Fillings more than 2 mm short from the apex were more frequent in the NTG than in the ATG. Almost one third (31.86%) of the natural teeth had fillings unsatisfactorily placed more than 2 mm from the apex; such occurrences happened in 11.27% of the artificial teeth. All the artificial teeth had a patent foramen, while natural teeth were subject to obliteration and curvature, resulting in non‐patency or difficult to reach patency. This aspect could have favoured the results for artificial teeth. It seems that the overall quality of the fillings was unsatisfactory. However, it must be pointed out that the second‐year students in this study had low dexterity; a recent systematic review showed satisfactory fillings occurred in only 48% of the cases [14].

Ledges and root fractures were more frequent in the ATG (8.33%) than in the NTG (2.94%), which is similar to a recent clinical study [13]. The reciprocating kinematics are known to be safe, especially when inexperienced operators are evaluated [15, 16]. We can infer that the softness of the material resulted in deviation from the path even in straight canals. In other cases, the filling could be short from the apex, and the deviation was a result of the melted resin during Tagger's technique of obturation. A similar thought process can be applied for root fractures. The occurrence of root fractures in the artificial teeth (5.88%) was considerably higher than the in vivo prevalence [17]. However, the pattern of some fractures, in an axial direction, did not replicate the clinical setting [18]. Both occurrences were informed to the manufacturer, and improvement of the material was suggested.

Instrument separation was similar in both groups and within the findings of clinical studies [19]. Therefore, the artificial teeth could reliably mimic the natural teeth in this aspect. It is worthwhile to mention that a couple of instrument separations in the natural teeth were due to poor case selection, which is a main drawback when using natural teeth for preclinical training [20]. The lack of standardisation and difficulty of obtaining natural teeth resulted in students using severely curved canals for their training. A previous study showed no instrument separation by undergraduate students with both rotary and reciprocating kinematics [21]. In that previous study, assessing clinical cases performed by third‐ and fourth‐year students, case selection was strictly conducted, preventing accidents in patients.

A second aim of the present study was to interview the students and obtain their perceptions about the preclinical training. The null hypothesis 2 is that both groups have the same perception of the training. This hypothesis was also rejected. The 10‐question questionnaire involved ethical and technical aspects of the natural and artificial teeth. Fifty percent of the students thought that obtaining natural teeth was easy, and 91.67% of them stated that the teeth were obtained from banks or donations from private practice; meanwhile, 8.33% of the students could not state the origin of the teeth. Most of the students of the ATG found it easy to buy the teeth; moreover, 66.66% of the students responded that it would be difficult to obtain natural teeth if they were required to do so.

Both groups found that their assessment was fair in comparison with their colleagues. This is a surprising finding because in the NTG the anatomical variability among the students was considerable. For the ATG it was expected due to the similar aspects of the teeth.

Natural teeth can represent a risk of contamination if not properly managed [22]. It is also worthwhile to mention that the students in the NTG were less concerned about the biological risk and the ethical aspect of the natural teeth. This might be explained by the fact that natural teeth are largely used in dentistry for educational purposes. The students who used natural teeth stated that these teeth closely simulated the clinical aspect of access, shaping, irrigation and obturation. When the groups were compared, the students who used natural teeth had a better experience with shaping and irrigation when compared with their colleagues of the artificial teeth. Therefore, it is not a surprise that most of both groups would prefer to complete their training with natural teeth.

The limitations of the present study are clear. Two different classes were used for the evaluation; therefore, differences of interest and ability between the groups could impact the results. However, the same instrumentation and filling techniques were taught to the students. There was no difference in the faculty members and the same laboratory was used for both groups. Moreover, the same theoretical content was delivered for both groups. Similarly, to Tchorz et al. [23] we suggest that future studies should compare the clinical outcomes of the students in their clinical activities.

It can be concluded that the LikeReal teeth, when compared to natural teeth, presented more ledges and root fractures; however, less damage to the marginal ridge and short filling was observed. Students' perception was that natural teeth are more appropriate for preclinical training.

## Conflicts of Interest

The authors declare no conflicts of interest.

## Data Availability

Data generated in this study are available from the corresponding author upon reasonable request with a completed Materials Transfer Agreement, excluding the materials including personally identifiable information.
